# Functional autoantibodies against G-protein coupled receptors in patients with persistent Long-COVID-19 symptoms

**DOI:** 10.1016/j.jtauto.2021.100100

**Published:** 2021-04-16

**Authors:** Gerd Wallukat, Bettina Hohberger, Katrin Wenzel, Julia Fürst, Sarah Schulze-Rothe, Anne Wallukat, Anne-Sophie Hönicke, Johannes Müller

**Affiliations:** a Experimental and Clinical Research Center, Charité Campus Buch, Max-Delbrück Center for Molecular Medicine, Berlin, Germany; b Berlin Cures GmbH, Berlin; Germany; c Department of Ophthalmology, University of Erlangen, Friedrich-Alexander-University of Erlangen-Nürnberg, Erlangen, Germany; d Department of Medicine 1, University Hospital Erlangen, Erlangen, Germany

**Keywords:** Autoantibody, Autoimmunity, COVID-19, Fatigue, Post-covid-19 symptom, Long-COVID, _f_AAB, Functional autoantibody, ACE2, Angiotensin-converting enzyme 2 receptors, α_1_-_f_AAB, Autoantibody targeting the alpha1-adrenoceptor, AT1-_f_AAB, Autoantibody targeting the angiotensin II AT1 receptor, β_2_-_f_AAB, Autoantibody targeting the beta2-adrenoceptor, CRPS, Complex regional pain syndrome, ETA-_f_AAB, Autoantibody targeting the endothelin receptor, GPCR, G-protein coupled receptors, MAS-_f_AAB, Autoantibody targeting the MAS receptor, M_2_-_f_AAB, Autoantibody targeting the muscarinic receptor, NOC-_f_AAB, Functionally active autoantibody against the nociceptin receptor, PoTS, Postural orthostatic tachycardia syndrome, SARS, Severe acute respiratory syndrome, RAS, Renin angiotensin system

## Abstract

Impairment of health after overcoming the acute phase of COVID-19 is being observed more and more frequently. Here different symptoms of neurological and/or cardiological origin have been reported. With symptoms, which are very similar to the ones reported but are not caused by SARS-CoV-2, the occurrence of functionally active autoantibodies (_f_AABs) targeting G-protein coupled receptors (GPCR-_f_AABs) has been discussed to be involved.

We, therefore investigated, whether GPCR-_f_AABs are detectable in 31 patients suffering from different Long-COVID-19 symptoms after recovery from the acute phase of the disease.

The spectrum of symptoms was mostly of neurological origin (29/31 patients), including post-COVID-19 fatigue, alopecia, attention deficit, tremor and others. Combined neurological and cardiovascular disorders were reported in 17 of the 31 patients. Two recovered COVID-19 patients were free of follow-up symptoms. All 31 former COVID-19 patients had between 2 and 7 different GPCR-_f_AABs that acted as receptor agonists. Some of those GPCR-_f_AABs activate their target receptors which cause a positive chronotropic effect in neonatal rat cardiomyocytes, the read-out in the test system for their detection (bioassay for GPCR-_f_AAB detection). Other GPCR-_f_AABs, in opposite, cause a negative chronotropic effect on those cells. The positive chronotropic GPCR-_f_AABs identified in the blood of Long-COVID patients targeted the β_2_-adrenoceptor (β_2_-_f_AAB), the α_1_-adrenoceptor (α_1_-_f_AAB), the angiotensin II AT1-receptor (AT1-_f_AAB), and the nociceptin—like opioid receptor (NOC-_f_AAB). The negative chronotropic GPCR-_f_AABs identified targeted the muscarinic M_2_-receptor (M_2_-_f_AAB), the MAS-receptor (MAS-_f_AAB), and the ETA-receptor (ETA-_f_AAB). It was analysed which of the extracellular receptor loops was targeted by the autoantibodies.

## Highlights

• Sera from Long-COVID syndrome patients contained functionally active autoantibodies targeting G-protein coupled receptors.
• Autoantibodies target β_2_- and α_1_-adrenoceptors, angiotensin II AT1-, muscarinic M_2_-, MAS-, nociceptin- and ETA-receptors.
• Included syndromes were of neurological and cardiological origin, or a combination of both.
• Such autoantibody patterns have previously been seen in COVID independent neurological deficits and cardiovascular disease.

## 1 Introduction

The pandemic COVID-19 viral infection is often associated with severe respiratory and neurological complications, cardiovascular problems, microvascular and endothelial disorders, and gastrointestinal diseases. Additionally, these symptoms are often observed in patients who have already recovered from the disease and had negative follow-up coronavirus tests. In their Italian study, Carfi et al. [1] indicated that only 12.6% of investigated patients did not develop any persistent symptoms after recovering from COVID-19. Most of the symptomatic post-infection COVID-19 patients suffered from neurological disorders, such as chronic fatigue syndrome, postural orthostatic tachycardia syndrome (PoTS) and dysautonomia [1]. However, other neurological diseases, such as transverse myelitis, acute necrotising myelitis, Guillain-Barré syndrome and others, have also been reported in several recent case reports on patients following SARS-CoV-2 infection [[2], [3], [4], [5], [6], [7], [8], [9]]. Similar results concerning the extent of post-COVID-19 symptoms were also obtained in a German study that showed only 22% of their investigated COVID-19 patients stayed free of post-disease symptoms [10].

Besides neurological manifestations, patients who recovered from COVID-19 also often developed cardiovascular implications [11]. The most prevalent abnormalities observed included myocardial inflammation, arrhythmia, tachycardia, bradycardia, and atrioventricular (AV) block [10,[12], [13], [14], [15], [16], [17], [18]]. In a large multi-centre study, including the intensive care units of 68 geographically diverse hospitals across the United States, Hayek and co-workers investigated 5019 critically ill COVID-19 patients. They observed that of these 5019 patients, 14% (701/5019) had an in-hospital cardiac arrest which was associated with poor survival, particularly among older patients [19].

Several authors assumed that autoimmune processes, involving the formation of autoantibodies, may be involved in the pathogenesis [20] and development of a post-COVID-19 syndrome [[20], [21], [22]]. In an initial study by Zhou et al. [20], 21 patients critically ill with COVID-19 were investigated for the presence of autoantibodies. It was found that 20% had anti-52 kDa SSA/Ro antibodies (autoantibodies against extractable nuclear antigens), 25% had anti-60 kDa SSA/Ro antibodies and 50% had anti-nuclear antibodies.

Other autoantibodies acting as drivers of the disease have also been reported. It was recently shown by Bastard et al. that over 10% of their investigated COVID-19 patients with a life-threatening pneumonia condition (n ​= ​987) presented with neutralizing autoantibodies against interferon-ω (IFN-ω, n ​= ​13), the 13 types of IFN-α (n ​= ​36), or against both (n ​= ​52). A few of their patients also showed autoantibodies against the other three type I IFNs. In contrast, the authors did not see any of these autoantibodies in 663 patients with asymptomatic or mild SARS-CoV-2 infection and they were only present in 4 of 1227 healthy subjects included for comparison [23]. Bastard et al. concluded from their data that the pre-existence of neutralizing anti-type I IFN autoantibodies was the cause of a critical condition, rather than it being the consequence of the infection [23].

Novelli et al. concluded from their comprehensive systematic review about chronic inflammatory and autoimmune diseases onset during COVID-19 that “*it is likely than the autoimmune manifestations described in COVID-19 represent more the results of the inflammatory cascade and the immune activation triggered by the virus rather than a direct effect of the virus per se*” [24].

In another study, Lyons-Weiler compared immunogenic peptides of SARS-CoV-2 with sequences of human proteins and found a high number of matching homologous sequences [25]. This would explain the high rate of persisting autoreactivity after SARS-CoV-2 infection. Kreye et al. examined neutralizing anti-SARS antibodies of isolated B-cell clones and observed that some showed “self-reactivity” while others were virus neutralizing only without showing any self-reactivity [26]. The possible impact of autoantibodies on the pathogenesis has most recently been discussed by Khamsi [27].

It is a proven fact that autoimmune processes and the formation of functional autoantibodies (_f_AABs) directed against G-protein coupled receptors (GPCR) play a role in the development of neurological [[28], [29], [30]] and cardiovascular symptoms [31]. Therefore, in this present study, it was tested if such GPCR-_f_AABs might also be associated with the development of corresponding post-COVID-19 symptoms. We investigated virus-free sera from 31 recovered COVID-19 patients with respect to the occurrence of GPCR-_f_AABs.

## 2 Material and methods

### 2.1 Patient sera

Sera were obtained from 31 patients, 29 who were still suffering from post-COVID-19 symptoms, after recovery from acute disease and 2 patients who were symptom-free (all positive tested by PCR). All patients signed a written informed consent form which included giving permission to include the anonymised clinical data in a scientific publication, in agreement with the Declaration of Helsinki. Using the RedCap project for data collection and management [32] with the permission from the ethics commission (no: 295-20 ​B), 6 of the sera were recruited at the University of Erlangen.

### 2.2 Serum

As a safety-precaution, the COVID-19 patient sera were heat inactivated for 30 ​min at 56 ​°C before use. Afterwards, 0.4 ​mL of the samples were dialysed against 1 ​L of dialysing buffer (0.15 ​M NaCl, 10 ​mM phosphate buffer, pH 7.4; Membra-Cel MD 44, 14 ​kDa, Serva) for 24 ​h to remove low-molecular weight bioactive compounds and peptides. Finally, 40 ​μL of the dialysed samples were added to the bioassay (final dilution of 1:50).

### 2.3 Bioassay for measurement of GPCR-_f_AABs

For the identification and characterisation of GPCR-_f_AABs, a bioassay was used, as described in great detail by Davideit et al. [33] and Wenzel et al. [34] for GPCR-_f_AABs against the beta1-adrenoceptor, and for other GPCR-_f_AABs by Wallukat et al. [30,35,36]. After contact with the respective autoantibodies, a change in basal beating rate of spontaneously beating cardiomyocytes expressing GPCR was used as the measuring signal. The receptor specificity was checked by either subsequent addition of specific receptor blockers, resulting in an annulation of this effect, or by addition of corresponding receptor-epitope-competing extracellular loop peptides. In detail: for the specification of the β_2_-_f_AABs, the receptor antagonist ICI118.551 (0.1 ​μM) was used and also neutralizing peptides corresponding to the first (HILMKMWTFGNFWCEFWT) or second (HWYRATHQEAINCYANETCCDFFTNQ) extracellular loop of the human β_2_-adrenoceptor. The effect of the negative chronotropic muscarinic M_2_ receptor-autoantibody (M_2_-_f_AAB) was blocked by atropine (1 ​μM). Losartan (1 ​μM) blocked the effect of the positive chronotropic AT1-_f_AAB and A779 (1 ​μM) blocked the effect of the negative chronotropic MAS-_f_AAB. For the identification of the MAS-_f_AAB, additional competing peptides corresponding to the first and second extracellular loop of the human MAS receptor of the following sequences: LSIDYALDYELSSGHHYTIVTL and LSGEESHSRSDCRAN, respectively, were exploited. ETA-_f_AABs were identified by blocking their negative chronotropic effects through the addition of the specific endothelin receptor antagonist BQ123 (0.1 ​μM) and also competing peptides corresponding to the first or second extracellular loop of the receptor of the following sequences, LPINVFKLLAGRWPFDHNDFGVFLCKL and FEYRGEQHKTCMNATSKFMEFYQDVKD, respectively. The nociceptin receptor antagonist J113397 (0.1 ​μM) was used to block the effects of the positive chronotropic NOC-_f_AAB and also competing peptides corresponding to the first (LAVCVGGLLGNCLVMYV) or second (FTLTAMSVDRYVAICHPIRALDVR) extracellular loop. Addition of 1 ​μM urapidil or prazosin abolished the positive chronotropic effect of α_1_-_f_AABs. For the loop analysis the following competing peptides corresponding to the 1^st^ or 2^nd^ extracellular loop of the receptor were used: LGYWAFGRVFCN and GWRQPAPEDETICQINEEPGYVLFSAL, respectively. For all peptides 2 ​μL of a stock solution of 100 ​μg/mL was added to 40 ​μL of the corresponding GPCR-_f_AAB sample and incubated for 30min before the mixture was transferred to the cells.

## 3 Results

Several different GPCR-_f_AABs were identified in the 31 sera of recovered COVID-19 patients. All 31 investigated patients had between 2 and 7 different GPCR-_f_AAB (Table 1). This was a surprising unexpected effect. In healthy controls, which are included in many studies, these autoantibodies are only found in a small percentage [37,38]”.

Two functionally active autoantibodies, that were seen in almost all investigated former COVID-19 patients, were directed against the β_2_-adrenoceptor (β_2_-_f_AAB) and the muscarinic M_2_ receptor (M_2_-_f_AAB). These _f_AABs induced a positive and a negative chronotropic response on their targeted receptors, respectively.

Two other _f_AABs that were also present in 29 (90%) of the 31 investigated post-COVID-19 patients were directed against the angiotensin II AT1 receptor (_f_AT1-AAB) and the angiotensin 1-7 MAS receptor (MAS-AAB). These receptors belong to the renin angiotensin system (RAS) and cause a positive and negative chronotropic effect, respectively, when targeted by the respective _f_AABs.

Post-infection hair loss (alopecia) was experienced by 8 of the recovered patients. In sera of these patients, three additional GPCR-_f_AABs were discovered: the negative chronotropic ETA-_f_AAB (4/8), the positive chronotropic NOC-_f_AAB (5/8), and the positive chronotropic α_1_-AAB (3/8). Not every alopecia patient showed all three of these GPCR-_f_AABs. Instead, their occurrence varied, and a pattern is not yet detectable. As shown in (Table 1), 2 of the 31 investigated post COVID-19 patients developed _f_AABs without showing any symptoms.

**Table 1:** Overview of post-COVID-19 symptoms and accompanying GPCR-_f_AABs.

Patient no.	Gender	Age (years)	Running no.	Symptom class		Symptoms		Neuro-active _f_AABs	Vasoactive _f_AABs			Neuro- and vasocative _f_AABs	RAS-specific _f_AABs	
				Neuro∗	Cardiovasc∗∗	Neuro∗	Cardiovasc∗∗	Noc-_f_AAB^§^	β_2_-_f_AAB^$^	α_1_-_f_AAB^&^	ETA-_f_AAB^+^	M_2_-_f_AAB^%^	AT1-_f_AAB^?^	MAS-_f_AAB^#^
1	F	48	1	x	x	Fatigue, Alopecia, Anomic aphasia	Tachycardia	x	x		x	x	x	x
7	F	55	2	x	x	Fatigue, Alopecia	Tachycardia	x	x	x	x	x	x	x
11	F	39	3	x	x	Fatigue, Alopecia	Tachycardia	x	x			x	x	x
19	F	34	4	x	x	Fatigue, PoTS, Tremor	Tachycardia		x	x		x	x	x
22	F	34	5	x	x	Fatigue, Alopecia	Tachycardia		x	x		x	x	x
29	F	49	6	x	x	PoTS	Tachycardia		x	x		x	x	x
26	M	28	7	x	x	PoTS	Tachycardia, Hypertension	x	x			x	x	x
30	M	55	8	x	x	PoTS	Bradycardia		x			x	x	x
27	M	69	9	x	x	PoTS, Attention deficit	Tachycardia	x	x			x	x	x
31	M	44	10	x	x	Attention deficit	Bradycardia		x			x	x	x
3	F	56	11	x	x	Fatigue, Attention deficit	Tachycardia,	x	x		x	x	x	x
21	F	28	12	x	x	Attention deficit, Tremor, Dysautonomia	Arrhythmia		x			x	x	x
18	F	53	13	x	x	Tremor, Attention deficit	Tachycardia		x			x		
20	M	54	14	x	x	Attention deficit	Tachycardia, Hypertension						x	x
14	F	57	15	x	x	Fatigue, Anomic aphasia	Arrhythmia, Hypertension	x	x			x	x	x
23	F	50	16	x	x	Eczema, Alopecia	Myocarditis		x		x	x	x	x
28	M	65	17	x	x	Smell/Taste disorder.	Tachycardia, Myocarditis	x	x			x	x	x
24	F	33	18	x	x	Fatigue, PoTS	n.a.	x	x	x		x	x	x
2	M	42	19	x	–	Fatigue, Alopecia	n.a.		x			x	x	x
4	M	50	20	x	–	Fatigue	n.a.		x			x	x	x
5	F	45	21	x	–	Fatigue	n.a.		x			x	x	x
6	F	36	22	x	–	Tremor, Alopecia, Dysautonomia	n.a.	x	x	x	x	x	x	x
9	F	50	23	x	–	Fatigue	n.a.	x	x			x	x	x
10	F	48	24	x	–	Fatigue	n.a.		x		x	x	x	x
12	F	53	25	x	–	Fatigue, Attention deficit	n.a.	x	x			x	x	x
15	F	46	26	x	–	Fatigue, Alopecia, Polyneuropathy	n.a.	x	x			x	x	x
17	F	49	27	x	–	Fatigue, PoTS, Tremor	n.a.		x			x		
25	F	58	28	x	–	Attention deficit, Neuropathy	n.a.		x		x	x	x	x
13	F	26	29	x	–	Fatigue	n.a.		x			x	x	x
8	M	71	30	–	–	Symptom free	Symptom free		x	x	x	x	x	x
16	M	54	31	–	–	Symptom free	Symptom free	x	x			x	x	x

Neuro∗ ​= ​neurological symptoms; Cardiovasc∗∗ ​= ​cardiovascular symptoms, n.a. ​= ​not applicable, PoTS ​= ​postural orthostatic tachycardia syndrome; NOC-_f_AAB^§^ ​= ​functionally active autoantibody against the nociceptin receptor, β_2_-_f_AAB^$^ ​= ​autoantibody targeting the beta_2_-adrenoceptor, α_1_-_f_AAB^&^ ​= ​autoantibody targeting the alpha1-adrenoceptor, ETA-_f_AAB^+^ ​= ​autoantibody targeting the endothelin receptor, M_2_-_f_AAB^%^ ​= ​autoantibody targeting the muscarinic receptor, AT1-_f_AAB^?^ ​= ​autoantibody targeting the angiotensin II AT1 receptor, MAS-_f_AAB^#^ ​= ​autoantibody targeting the MAS receptor.

**Fig. 1:**
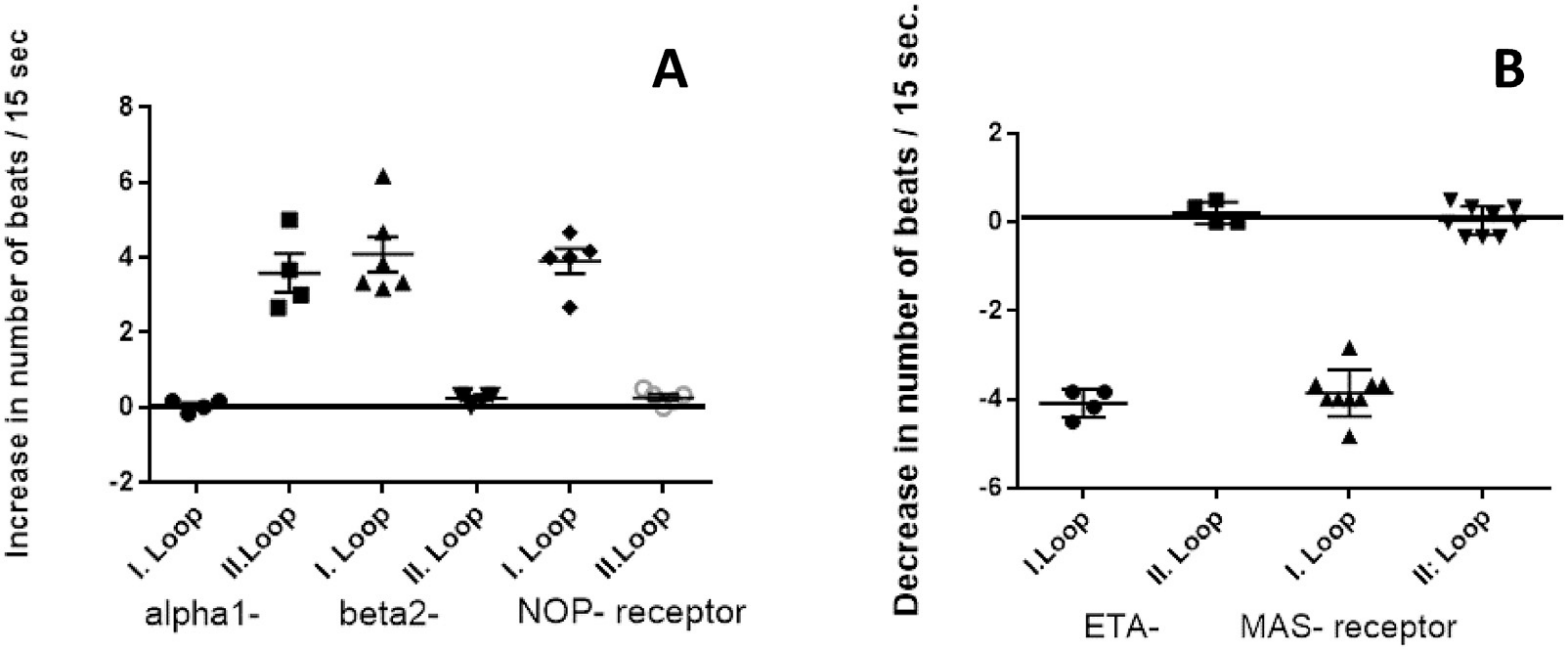
**Loop analysis of selected GPCR-**_**f**_**AAB positive samples. A:** positive chronotropic GPCR-_f_ABBs: α_1_-_f_AAB, β_2_-_f_AAB and NOC-_f_AAB and **B:** negative chronotropic GPCR-_f_ABBs: ETA-1-_f_AAB, MAS-_f_AAB samples were preincubated with 0.2 ​μg of the corresponding competing loop peptides as indicated under Material and methods for 30 ​min before the mixture was added to the cells for the recording of the corresponding GPCR-_f_AAB effect. In the case of competition, the chronotropic response was abolished, which was achieved in four cases by the loop peptides specific to the 2nd extracellular loops. Only the α_1_-_f_AAB targeted the 1^st^ extracellular loop.

In a next step it was analysed at selected ETA-1-_f_AAB, MAS-_f_AAB and α_1_-_f_AAB, β_2_-_f_AAB and NOC-_f_AAB positive samples which of the extracellular loops of the receptors were targeted by the respective autoantibodies by addition of competing loop peptides (Fig. 1). Here it was clearly to see that with four of the tested autoantibodies (ETA-1-_f_AAB, MAS-_f_AAB, β_2_-_f_AAB, and NOC-_f_AAB) in each of the investigated cases the second extracellular loop of the receptor was targeted by the autoantibody and consequently neutralized by addition of its competing loop peptide. α_1_-_f_AAB containing samples targeted the 1^st^ extracellular loop of the receptor. This analysis provided additional evidence that no nonspecific effects had influenced the outcome.

## 4 Discussion

The astonishing finding of this investigation is the fact that an unusually high number of GPCR-_f_AABs were detected in the serum of recovered COVID-19 patients who mostly suffered from a variety of different post-COVID-19 symptoms. Due to the functionality of such GPCR-_f_AABs, the question of whether these GPCR-_f_AABs may play a role in the development of post-COVID-19 symptoms is raised.

A continuing fatigue-like symptom, persisting long after virus follow-up tests are negative, was a frequently reported impairment in patients of this study (17/31), and other studies [1]. For patients suffering from a classical coronavirus-independent fatigue syndrome, the occurrence of β_2_-_f_AABs, M_2_-_f_AABs and, in some cases, also ETA-_f_AABs has already been reported before [39]. Here, with this post-COVID-19 study, almost all investigated sera contained β_2_-_f_AABs and M_2_-_f_AABs. The combination of β_2_-_f_AABs and M_2_-_f_AABs have also been identified in sera of patients suffering from PoTS and dysautonomia [40], both of which are conditions now observed in post-COVID-19 patients (7/31 and 2/31, respectively, not overlapping). Furthermore, this combination of β_2_-_f_AABs with M_2_-_f_AABs had also been identified before by our group, in patients with complex regional pain syndrome (CRPS) [41], in patients suffering from narcolepsy type 1, here additionally with the NOC-_f_AAB in 9 of 10 cases [36] and in patients with small fibre diseases.

Two of the identified GPCR-_f_AABs, observed in over 90% of the investigated COVID-19 patient sera (29/31), were directed against receptors of RAS, namely the angiotensin II AT1 receptor and the angiotensin (1–7) MAS receptor. These vasoactive AT1-_f_AABs had been identified before in patients with malignant hypertension, therapy-resistant hypertension, preeclampsia, and kidney diseases [[42], [43], [44]]. Moreover, Dragun et al. [44] showed that AT1-_f_AABs induced the rejection of kidneys in a subgroup of patients that underwent kidney transplantation. The authors also showed that the transfer of human AT1-_f_AABs to kidney transplanted rats caused the occlusion of the kidney arteries in the recipients.

Given the evidence described above, it may be that vasoactive processes, caused by the occurrence of AT1-_f_AABs and MAS-_f_AABs, might also be involved in the pathogenesis of post-COVID-19 symptoms. However, it is highly unlikely that the pathophysiological effects are caused by the _f_AABs alone. For example, Lukitsch and co-workers [45] already showed that the addition of the human AT1-_f_AABs to isolated kidney arteries induced a contraction of these arteries, but only in ischemic arteries and in arteries that were taken from kidney transplanted rats. Arteries obtained from healthy rats did not respond to AT1-_f_AABs, even though they reacted to their natural agonist, angiotensin II (which confirmed that the receptors were intact). Taken together, these data demonstrated that the AT1-_f_AABs did not act alone but needed ischaemic or inflammatory cofactors to have full effect. With respect to the COVID-19 situation, this is of course an absolutely obvious situation. Thrombo-inflammatory factors which even may become predictive markers for COVID-19 complications have been described by Cremer et al. [46]. Other immune biomarkers, as taken together by Fouladseresht et al. [47] have also been reported.

To date, the evidence suggests that a combination of ischaemic or inflammatory cofactors and autoantibodies can act to maintain a cardiac inflammatory process. Specifically, it has been shown that AT1-_f_AABs and α1-_f_AABs can influence the maturation and degranulation of cardiac mast cells [48], suggesting that they can contribute to inflammation.

It has also already been reported that COVID-19 induces an imbalance of RAS through viral-occupation of the angiotensin-converting enzyme 2 (ACE2) receptors, which reduces the generation of protective-peptides angiotensin-(1–7) and (1–9). This subsequently decreases the stimulation the MAS- and angiotensin II ATR2-receptors, and is accompanied by an overstimulation of the AT1-receptors due to reduced degradation of angiotensin II by ACE2 [49]. Therefore, Steckelings and Sumners [49] recently suggested that ATR2 receptor agonists could be used to treat COVID-19-induced disorders of various organ systems.

This is in good agreement with the identification of MAS-_f_AABs in over 90% of the symptomatic patients examined. Autoantibodies against this receptor have been observed before in a cancer patient after chemotherapy [50] and in patients with multiple sclerosis (MS) (unpublished results). In MS patients we observed the combination of MAS-_f_AABs with α_1_-_f_AABs. In this context, it is interesting to see that in several case reports it was shown that COVID-19 patients can develop neurological complications like transverse myelitis [2] and Guillain-Barré syndrome [5,7]. Whether the MAS-_f_AABs observed in this COVID-19 study is involved in the development of the reported neurological symptoms should be clarified in further investigations.

Furthermore, our data showed that 2 of the patients with a mild COVID-19 infection developed _f_AABs but not the symptoms as seen in the other recovered COVID-19 patients. We assume that in both of these patients adaption processes might have prevented the binding of _f_AABs to the receptor or the receptors are not available for the _f_AABs as described by Lukitsch and co-worker for angiotensin II AT1 _f_AAB before [45].

We strongly assume that the GPCR-_f_AABs play an important role in the development and maintenance of post-COVID symptoms. These GPCR-_f_AABs persistently stimulate their corresponding receptors and the normal, physiological, cell-protective desensitisation of the receptors is inhibited by the _f_AABs themselves [51].

It has already been shown in other diseases, such as idiopathic dilated cardiomyopathy (here it is the autoantibody targeting the beta1-adrenoceotor), that GPCR-_f_AABs play a significant role in the pathogenesis of the disease [31]. Additionally, the removal of these _f_AABs by immunoadsorption led to an improvement in cardiac function and to a significant increase in survival rate [52,53]. A similar beneficial therapeutic effect of the removal of GPCR-_f_AABs has also been observed in β_2_-_f_AAB positive, therapy-refractory, open-angle glaucoma patients. Here, the removal of the _f_AABs by immunoadsorption resulted in a reduction of the ocular pressure [54].

With respect to post-COVID-19 symptoms, Masuccio et al. reported a patient who was suffering from post-infection acute motor axonal neuropathy and myelitis. The patient tested positive for the ganglioside anti-GD1b IgM autoantibody, and a partial recovery was achieved through plasma exchange combined with subsequent immunoglobulin substitution [7].

The Sars-CoV-2 spike protein is a potential epitopic target for biomimicry-induced autoimmunological processes [25]. Therefore, we feel it will be extremely important to investigate whether GPCR-_f_AABs will also become detectable after immunisation by vaccination against the virus.

## 5 Conclusion

Our results indicated that all 29 investigated symptomatic post-COVID-19 patients developed _f_AABs directed against different GPCRs, known to be able to disturb the balance of neuronal and vascular processes. Most of these patients developed an antibody pattern consisting of β_2_-_f_AABs, M_2_-_f_AABs, AT1-_f_AABs, and MAS-_f_AABs. These agonistic _f_AABs activate their corresponding receptors like classical agonists. The observed specific GPCR-_f_AAB pattern has been observed before in several neurological and cardiac disorders and might also support the development of neurological and/or cardiovascular symptoms after COVID-19 recovery. These results provide valuable clues that are worth pursuing and investigating further.

## 6 Limitations of the study

A major limitation of this study is that it is only a snapshot. Therefore, the causal relationship between the presence of GPCR-_f_AAB and the disease cannot be shown. In order to be able to identify causal relationships, samples from former COVID patients must be systematically collected over a longer period of time for GPCR-_f_AAB detection. Afterwards the data have to be retrospectively assigned to recovering and non-recovering Long-COVID-19 patients.

## Data availability

All available data are in included in this study.

## Ethical standard

Informed consent and permission to publish this information was obtained from every patient included in this study.

## Informed consent

Written informed consent was collected from the patients for the inclusion of anonymised clinical data in a scientific publication, in agreement with the Declaration of Helsinki.

## Compliance with ethical standards

.

## Funding

Part of this work was funded by the Berlin Cures GmbH, Germany. The funder provided support in the form of salaries but did not have any additional role in the study design, data collection and analysis, decision to publish, or preparation of the manuscript.

## Authors contributions

All authors contributed to the study conception and design. Material preparation and data collection and analysis were performed by Gerd Wallukat, Bettina Hohberger, Katrin Wenzel, Julia Fürst, Sarah Schulze-Rothe, Anne Wallukat, Anne-Sophie Hönicke, and Johannes Müller. The first draft of the manuscript was written by Annekathrin Haberland and all authors commented on previous versions of the manuscript. All authors read, revised, and approved the final manuscript.

## Declaration of competing interest

The authors declare the following financial interests/personal relationships which may be considered as potential competing interests. G. Wallukat, K. Wenzel, S. Schulze-Rothe, A. Wallukat, A.S. Hönicke, and J. Müller are employed by the Berlin Cures GmbH. G. Wallukat and J. Müller are shareholders of the Berlin Cures Holding AG, the holding company of Berlin Cures. The authors declare no competing interests. All other authors have nothing to declare. The authors have no other relevant affiliations or financial involvement with any organisation or entity with a financial interest in or financial conflict with the subject or materials discussed in the manuscript apart from those which are disclosed.
